# Clinical Outcome Predictive Value of Procalcitonin in Patients Suspected with Infection in the Emergency Department

**DOI:** 10.1155/2021/2344212

**Published:** 2021-06-10

**Authors:** Pierre Leroux, Sébastien De Ruffi, Laurent Ramont, Marion Gornet, Guillaume Giordano Orsini, Xavier Losset, Lukshe Kanagaratnam, Stéphane Gennai

**Affiliations:** ^1^Emergency Department, Reims University Hospital, 45 Rue Cognacq-Jay, Reims 51100, France; ^2^Biochemistry Department, Reims University Hospital, 45 Rue Cognacq-Jay, Reims 51100, France; ^3^Université de Reims Champagne-Ardenne, SFR CAP-Santé (FED 4231), Laboratoire de Biochimie Médicale et Biologie Moléculaire, 51 Rue Cognacq-Jay, Reims 51100, France; ^4^CNRS UMR 7369, Matrice Extracellulaire et Dynamique Cellulaire-MEDyC, 51 Rue Cognacq-Jay, Reims 51100, France; ^5^INSERM UMR-S1250, Pathologies Pulmonaires et Plasticité Cellulaire–P3Cell, 45 Rue Cognacq-Jay, Reims 51100, France; ^6^Clinical Research Unit, Reims University Hospital, 45 Rue Cognacq-Jay, Reims 51100, France

## Abstract

Procalcitonin (PCT) may be useful for early risk stratification in the emergency department (ED), but the transposition of published data to routine emergency practice is sometimes limited. An observational retrospective study was conducted in the adult ED of the Reims University Hospital (France). Over one year, 852 patients suspected of infection were included, of mean age 61.7 years (SD: 22.6), and 624 (73.2%) were hospitalized following ED visit. Overall, 82 (9.6%) patients died during their hospitalization with an odds ratio (OR) of 5.10 (95% CI: 2.19–11.87) for PCT ≥ 0.5, in multivariate logistic regression analyses. Moreover, 78 (9.2%) patients were admitted to an ICU, 74 (8.7%) had attributable bacteremia, and 56 (6.6%) evolved toward septic shock with an OR of 4.37 (2.08–9.16), 6.38 (2.67–15.24), and 6.38 (2.41–16.86), respectively, for PCT ≥ 0.5. The highest discriminatory values were found for patients with age <65 years, but PCT lost its discrimination power for in-hospital mortality in patients with a bronchopulmonary infection site or a temperature ≥37.8°C and for ICU admission in patients with severe clinical presentations. PCT could be helpful in risk stratification, but several limitations must be considered, including being sometimes outperformed by a simple clinical examination.

## 1. Introduction

The inflammatory biomarker procalcitonin (PCT) has shown some interest in infection diagnosis and antibiotic therapy guidance [1–4]. In the emergency department (ED), PCT may be useful for early risk stratification, as crowding has become a major issue, forcing emergency physicians (EPs) to make fast decisions, identifying both high risk/poor outcome and low risk/favorable outcome patients [4, 5]. Added to clinical presentation, PCT provides objective information about a patient's prognosis [2].

On the other hand, transposition of certain published studies to routine emergency practice can be limited, because the studies evaluated the interest of PCT with complete disconnection from the clinical presentation (including uninfected patients), [6–8] or focused on selected infected patients (e.g., patients with a particular infection site or infection type or infection severity level), [9–12] or targeted every infected patient regardless of the medical reasoning [13, 14]. In addition, numerous studies are issued from the same research teams, and some authors disclosed significant conflicts of interest (e.g., with laboratory manufacturing).

Systematic dosage of PCT is not a usual daily practice among EPs, who have the added concern of health care cost-effectiveness issues. PCT dosage should require a clinical justification, questioning its contribution to medical reasoning. Consequently, to better understand the clinical implication of PCT in patients suspected of infection, observational studies based on routine emergency practice are needed, as they reflect aspects of care that most randomized-control trials do not [15, 16].

Herein, we investigated, in a study based on routine emergency practice, the association between initial PCT levels, whose measurements have been purposefully prescribed by EPs in patients suspected of infection, and major clinical outcomes.

## 2. Materials and Methods

### 2.1. Aim and Outcomes

The first aim of this study was to investigate the association of initial PCT levels and major clinical outcomes in ED patients suspected of infection. The second aim was to analyze in-depth the discriminative power of PCT depending on demographic data, initial vital signs, and main infection sites.

Endpoints were in-hospital mortality, intensive care unit (ICU) admission, bacteremia, and septic shock, related to the same ED visit. In-hospital mortality was defined as the patient not surviving at hospital discharge. Only attributable bacteremia (related to the infection site, avoiding contaminations) was retained. Septic shock diagnosis was made according to the 2016 Surviving Sepsis Campaign criteria [17].

### 2.2. Design

An observational, retrospective, single-center study was conducted in the adult ED of the Reims University Hospital (France) from January 1^st^ to December 31^st^, 2018. All PCT measurements from the ED during this period were considered. Duplicates, dosages in patients under 18 years old, and patient files with relevant missing data were excluded. In a second time, patients with active malignancy or with the final noninfectious diagnosis were excluded as well.

Biological data were collected from the dedicated software of the biochemistry department (Haemonetics software). They included PCT, C-reactive protein (CRP), leukocytes, and neutrophil levels measured at the same time in the ED. PCT and CRP concentrations were performed with a Cobas 8000 biochemistry analyzer (Roche diagnostic). The serum concentrations of PCT were measured by Elecsys BRAHMA PCT electrochemiluminescence immunoassay, and the serum concentrations of CRP were determined by latex‐enhanced turbidimetric immunoassay according to the manufacturer's instructions. Leukocytes and neutrophils levels were measured with an ADVIA analyzer (Siemens-Healthineers). Blood cultures were carried out using a BACTALERT analyzer (Biomérieux). Clinical data were collected from digitalized patient files (Easily, SILPC) related to the same ED visit. They included demographic data, ED nurse triage level using the FRench Emergency Nurses Classification in Hospital scale (FRENCH) [18], initial vital signs, patient comorbidities, and the infection site. The final infectious disease diagnosis, the need for hospitalization, and the occurrence of the outcomes were further collected for each patient.

No specific recommendations were issued to physicians and nurses regarding ED patient management, before or during the study. Biomarkers were prescribed at the physician's discretion.

The study was performed in accordance with the Helsinki Declaration and met ethical requirements. The study was based on medical data systematically recorded at the Reims University Hospital and authorized by the French national commission for data privacy (Commission Nationale Informatique et Libertés, CNIL).

### 2.3. Statistical Analysis

The population was described using frequencies for qualitative data and mean with standard deviation (SD) or median with interquartile range (IQR) for quantitative data, as appropriate. The population was stratified into four individual groups with different PCT cut-offs (ng·mL^−1^) similar to the cut-offs proposed by Sager et al.: PCT < 0.1, 0.1 ≤ PCT < 0.25, 0.25 ≤ PCT < 0.5, PCT ≥ 0.5 [7]. Associations between initial PCT levels and sociodemographic, clinical, and biological data were investigated using the Chi-square or Fisher's exact test, Kruskal Wallis, or one-way analysis of variance, as appropriate.

The association between initial PCT levels and the four endpoints was investigated using univariate and multivariate logistic regression analyses, reporting odds ratios (OR) with 95% confidence intervals (CI) for each PCT group. The model was adjusted for age, gender, comorbidities, and infection site.

The discriminative power of PCT was assessed for each outcome, using the area under the receiver operating characteristics curve (AUC) analysis for the whole cohort and for selected subgroups stratified by gender, age, FRENCH level, temperature, systolic blood pressure (SBP), Glasgow Coma Scale (GCS) level, and infection site. Forest plots were used to present the data.

Analyses were performed using SAS, version 9.4 (SAS Institute, Inc., Cary, NC, USA).

## 3. Results

### 3.1. Population

In 2018, 1711 PCT assays were performed at the request of the ED, from which the following were excluded: 32 duplicates, 23 related to patients under 18 years old, and 21 related to patient files with relevant missing data. Of the remaining 1635 patients, 599 were excluded due to noninfectious final diagnosis and 184 because they presented an active malignancy. In the end, a total of 852 patients were included.

### 3.2. Patient Characteristics

The mean age of patients was 61.7 years (SD: 22.6), and 49.2% were male. The main acute infection sites leading to ED visits were bronchopulmonary in 31.0%, urogenital in 20.8%, intra-abdominal in 13.4%, skin and soft tissue in 8.8%, and undetermined in 15.6%. Other specified infection sites included ear, nose, throat, eye, tooth, cardiovascular, bone, and neurological infection sites. Diabetes was found in 24.7% and obesity in 13.6%. Among the 852 patients, 624 (73.2%) were hospitalized following ED visits. Patient characteristics stratified by the PCT group are shown in Table 1.

### 3.3. Association of Initial Procalcitonin Levels and Clinical Outcomes

Overall, 82 (9.6%) patients died during their hospitalization, with a stepwise increase in mortality rate from 0.8% to 5.6% of the cohort across predefined PCT level cut-offs (Table 2). This was confirmed by univariate and multivariate logistic regression analyses (adjusted for gender, age, comorbidity, and infection site), the association of PCT levels, and in-hospital mortality remaining significant, with an OR of 3.24 (95% CI: 1.22–8.63, *p*= 0.0185) for 0.25 ≤ PCT < 0.5, and 5.10 (2.19–11.87, *p*= 0.0002) for PCT ≥ 0.5.

Similar results were found for the other outcomes: during their hospital stay, 78 (9.2%) patients were admitted in an ICU, 74 (8.7%) had attributable bacteremia, and 56 (6.6%) evolved toward septic shock. In the fully adjusted model, the association of PCT levels ≥0.5 and the mentioned outcomes was also significant, with OR of 4.37 (2.08–9.16, *p* < 0.0001) for ICU admission, 6.38 (2.67–15.24, *p* < 0.0001) for bacteremia, and 6.38 (2.41–16.86, *p*=0.0002) for septic shock (Table 2).

### 3.4. PCT Discrimination for Clinical Outcomes, considering Specific Criteria

**Figure 1:**
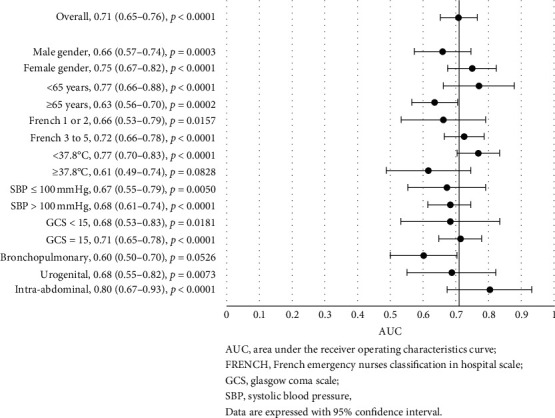
Forest plot of the area under the receiver operating characteristics curve for procalcitonin discrimination in in-hospital mortality, stratified by different criteria.

**Figure 2:**
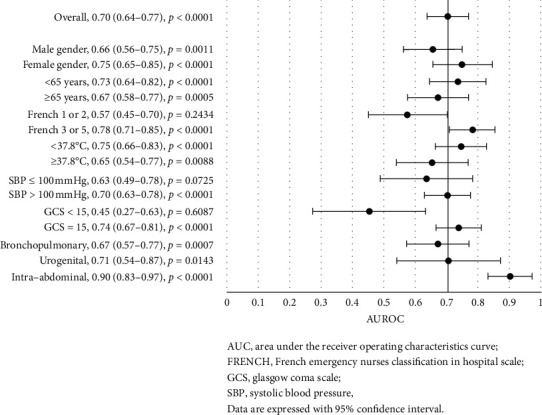
Forest plot of the area under the receiver operating characteristics curve for procalcitonin discrimination in ICU admission, stratified by different criteria.

**Figure 3:**
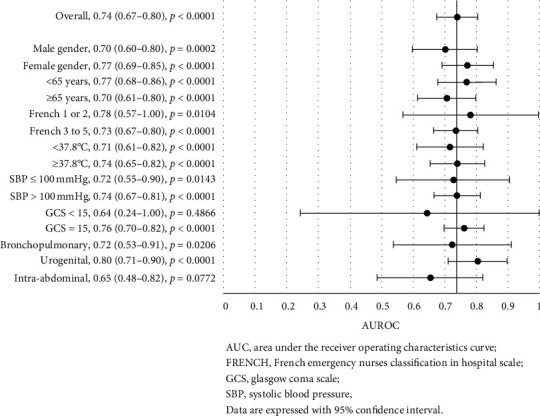
Forest plot of the area under the receiver operating characteristics curve for procalcitonin discrimination in bacteremia, stratified by different criteria.

**Figure 4:**
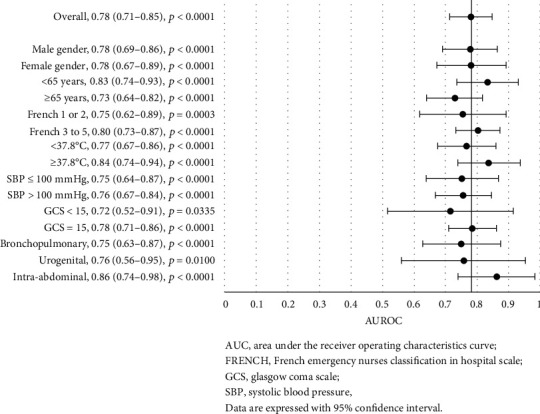
Forest plot of the area under the receiver operating characteristics curve for procalcitonin discrimination in septic shock, stratified by different criteria.

PCT showed good discrimination for in-hospital mortality with an area under the curve (AUC) of 0.71 (0.65–0.76, *p* < 0.0001) in the whole cohort (Figure 1). Similarly, PCT showed good discrimination for ICU admission with an AUC of 0.70 (0.64–0.77, *p* < 0.0001) (Figure 2), even better for bacteremia with an AUC of 0.74 (0.67–0.80, *p* < 0.0001) (Figure 3), and better for septic shock with an AUC of 0.78 (0.71–0.85, *p* < 0.0001) (Figure 4).

Further analyses of the prognostic accuracy of PCT were carried out across different subgroups. The highest discriminatory values were found for all the outcomes for patients with age <65, with AUC of 0.77 (vs 0.63 for patients with age ≥ 65) for in-hospital mortality, 0.73 (vs 0.67) for ICU admission, 0.77 (vs 0.70) for bacteremia, and 0.83 (vs 0.73) for septic shock. The comparison reached significance only for in-hospital mortality (*p*=0.0399). In addition, the highest discriminatory values were found for women, with AUC of 0.75 (vs 0.66 for men) for in-hospital mortality, 0.75 (vs 0.66) for ICU admission, and 0.77 (vs 0.70) for bacteremia, but not for septic shock (AUC: 0.78 vs 0.78). Conversely, PCT lost its discrimination power for in-hospital mortality in patients with a bronchopulmonary infection site (AUC: 0.60, *p*=0.0562) or with a temperature ≥ 37.8°C (AUC: 0.61, *p*=0.0828) (Figure 1). Intra-abdominal infection site was associated with higher PCT discriminatory values among the principal infection sites, for in-hospital mortality (AUC: 0.80), ICU admission (AUC: 0.90), and septic shock (AUC: 0.86), but not for bacteremia (AUC: 0.65, *p*=0.0772). For ICU admission, PCT lost its discrimination ability in patients with SBP <100 mmHg (AUC: 0.63*p*=0.0725), GCS < 15 (AUC: 0.45, *p*=0.6087) or rated FRENCH 1 or 2 (AUC: 0.57, *p*=0.2434) at the ED nurse triage (Figure 2).

## 4. Discussion

This first study based on routine emergency practice, investigating the prognostic value of PCT in ED patients suspected of infection, has 3 key findings. First, initial PCT levels ≥0.5 are highly associated with in-hospital mortality, ICU admission, bacteremia, and septic shock, independently of clinical parameters such as gender, age, comorbidity, and infection site, corroborating uneven recent findings [7, 8]. This association still exists between PCT levels ≥ 0.25 and in-hospital mortality. Second, PCT shows a good discriminating power for every studied outcome, even better in patients with age <65 (compared to older patients). Third, on the other hand, PCT loses its discriminating power in several clinical conditions: for in-hospital mortality in patients with a bronchopulmonary infection site, for bacteremia in patients with an intra-abdominal infection site, and for ICU admission in patients with clinical signs of severity.

As outlined, initial PCT values are not as effective at predicting certain major outcomes like mortality for older patients as for younger ones. To date, the prognosis value of PCT in elderly patients has been poorly investigated as the main objective. Even if some authors showed interesting prognosis value of PCT for bacteremia in patients ≥65 years presenting a systemic inflammatory response syndrome [19], some others highlighted several limitations while using PCT as a prognosis biomarker in elderly patients. [20–22]. Steichen et al. concluded that despite a good overall diagnostic accuracy, the clinical usefulness of PCT to diagnose invasive bacterial infections in patients ≥75 years was limited [20]. Kim et al. showed no association between PCT levels and mortality in patients ≥65 years presenting community-acquired pneumonia [21]. Similarly, Lee et al. found no effectiveness for PCT in predicting mortality in patients ≥65 years with sepsis [22]. Such mitigated findings probably result from the higher rate of comorbidities in the older population.

Like others [7], we found higher initial PCT rates in older patients than younger ones. It is unclear if this difference reflects a higher rate of serious infections in older patients or a lower clearance of PCT blood levels in this population, or both, and if PCT could have a negative impact on patients' medical conditions, as mortality increased with an injection of PCT and decreased with PCT neutralization in animal models [23, 24].

Interestingly, we found no initial PCT level ability in predicting in-hospital mortality for patients with a bronchopulmonary infection. This finding is somewhat surprising, since the respiratory tract, as the most common infection site, has motivated extensive research on PCT. Yet, in this context, PCT was essentially used for antibiotic stewardship. There is poor research on PCT pathophysiological mechanisms, and no experimental research provides a clear explanation of this finding. One possible explanation could be the presence of a virus in more than 50% of acute respiratory tract infections [25]. Since viruses stimulate macrophage interferon-alpha synthesis, which inhibit tumor necrosis factor (promoter of PCT release) [26], PCT rates may be lowered in viral infections.

Conversely, initial PCT showed high discriminative power for in-hospital mortality, septic shock, and ICU admission, but not for bacteremia, in patients with an intra-abdominal infection site. Bacterial translocation triggered across the gut wall by gastrointestinal hypoperfusion [27] may partially explain this finding. Also, intra-abdominal infections may necessitate surgical cares (which remains unusual for bronchopulmonary infections), leading to an increase in morbimortality.

We also showed no discriminative power of PCT in ICU admission prediction in patients rated FRENCH 1 or 2 at ED nurse triage or with an SBP <100 mmHg or a GCS <15. This finding reflects the superiority of the semiology over PCT in predicting ICU admission, reducing the interest of its prescription for this purpose, in accordance with previous work [28].

This study has some limitations. First, we did not investigate the bacterial or viral etiology of each infection due to a lack of gold standard criteria. Second, we did not assess the cause of in-hospital mortality, and death may have not been directly related to the infection. Third, the study was designed on purpose to include ED patients suspected of infection for whom PCT was prescribed, setting aside other infected patients, and therefore limiting these results from being applied to all infected patients. Fourth, the retrospective nature of the study may have been the source of selection bias in the cohort of patients.

As an independent factor for major outcomes, PCT could be helpful in risk stratification in the ED. Patients with higher PCT levels may benefit from early antibiotic initiation and close monitoring. Yet, EPs must be aware of limitations in interpreting PCT levels in older patients and in patients with a bronchopulmonary or intra-abdominal infection site. Moreover, at times PCT may not be as useful in predicting ICU admission as a simple clinical examination.

## Figures and Tables

**Table 1:** Patient characteristics stratified by PCT group.

	Overall	PCT group (ng·mL^−1^)				*p* value
	*N* = 852	PCT < 0.1 *N* = 227	0.1 ≤ PCT < 0.25 *N* = 235	0.25 ≤ PCT < 0.5 *N* = 114	PCT ≥ 0.5 *N* = 276	
*Demographic*						
Male gender, *n* (%)	419 (49.2)	91 (40.1)	122 (51.9)	61 (53.5)	145 (52.5)	0.0161
Female gender, *n* (%)	433 (50.8)	136 (59.9)	113 (48.1)	53 (46.5)	131 (47.5)	
Age, mean (SD)	61.7 (22.6)	52.9 (22.3)	59.1 (23.2)	63.5 (22.7)	67.3 (20.2)	<0.0001

*Nurse triage level and vital signs*						
French 1 or 2, *n* (%)	90 (10.6)	23 (10.1)	23 (9.8)	11 (9.6)	33 (12.0)	0.8266
Temperature, °C, mean (SD)	37.5 (1.2)	37.2 (1.1)	37.6 (1.1)	37.6 (1.2)	37.8 (1.3)	<0.0001
SBP, mmHg, mean (SD)	128.4 (24.4)	131.2 (22.2)	131.7 (21.6)	131.1 (23.2)	122.3 (27.8)	<0.0001
DPB, mmHg, mean (SD)	73.0 (16.6)	76.4 (15.7)	73.9 (14.6)	74.2 (15.3)	69.1 (18.6)	<0.0001
Heart rate, bpm, mean (SD)	96.6 (23.0)	93.7 (22.0)	98.0 (22.7)	95.5 (21.5)	98.4 (24.6)	0.0981
Pulse oximetry, %, mean (SD)	94.1 (6.8)	95.3 (6.2)	94.3 (6.5)	93.5 (7.2)	93.4 (7.1)	<0.0001
GCS, mean (SD)	14.7 (1.4)	14.8 (1.0)	14.9 (0.8)	14.5 (2.0)	14.6 (1.7)	0.0508

*Comorbidities*						
Diabetes, *n* (%)	210 (24.7)	37 (16.3)	51 (21.7)	31 (27.2)	91 (33.0)	0.0001
Immunosuppression, *n* (%)	66 (7.8)	24 (10.6)	12 (5.1)	6 (5.3)	24 (8.7)	0.1040
Obesity, *n* (%)	116 (13.6)	24 (10.6)	32 (13.6)	16 (14.0)	44 (15.9)	0.3803

*Infection site*						<0.0001
Bronchopulmonary, *n* (%)	264 (31.0)	57 (21.6)	86 (32.6)	35 (13.3)	86 (32.6)	
Urogenital, *n* (%)	177 (20.8)	37 (20.9)	36 (20.3)	26 (14.7)	78 (44.1)	
Intra-abdominal, *n* (%)	114 (13.4)	27 (23.7)	40 (35.1)	14 (12.3)	33 (28.9)	
Skin and soft tissue, *n* (%)	75 (8.8)	27 (36.0)	13 (17.3)	9 (12.0)	26 (34.7)	
Other (specified), *n* (%)	89 (10.4)	35 (39.3)	16 (18.0)	12 (13.5)	26 (20.3)	
Undetermined, *n* (%)	133 (15.6)	44 (33.1)	44 (33.1)	18 (13.5)	27 (20.3)	

*Biology*						
CRP, mg·L^−1^, median (IQR)	63.7 (125.9)	16.6 (42.8)	50.5 (96.0)	89.7 (146.5)	133.0 (208.2)	<0.0001
Leukocytes, .10^9^ cells. L^−1^, median (IQR)	12.2 (7.8)	10.9 (6.1)	11.9 (6.3)	12.2 (7.6)	14.6 (10.2)	<0.0001
Neutrophils, .10^9^ cells. L^−1^, median (IQR)	11.0 (7.6)	8.2 (5.7)	9.5 (6.3)	10.0 (8.0)	12.7 (9.4)	<0.0001

CRP, C-reactive protein; DPB, diastolic blood pressure; FRENCH, French Emergency Nurses Classification in Hospital scale; GCS, Glasgow coma scale; IQR, interquartile range; PCT, procalcitonin; SBP, systolic blood pressure; SD, standard deviation.

**Table 2:** Association of initial procalcitonin levels and clinical outcomes in univariate and multivariate logistic regression models.

		Events *N* (%)	Unadjusted model OR (95% CI)	Adjusted model ‡ OR (95% CI)
*In-hospital mortality*		82 (9.6)		
PCT cut-offs	*p* < 0.0001			
PCT < 0.1		7 (8.5)	1	1
0.1 ≤ PCT < 0.25		14 (17.1)	1.99 (0.79–5.03), *p*=0.1451	1.70 (0.65–4.23), *p*=0.2773
0.25 ≤ PCT < 0.5		13 (15.9)	4.05 (1.57–10.45), *p*=0.0039	3.24 (1.22–8.63), *p*=0.0185
PCT ≥ 0.5		48 (58.5)	6.62 (2.93–14.94), *p* < 0.0001	5.10 (2.19–11.87), *p*=0.0002

*ICU admission*		78 (9.2)		
PCT cut-offs	*p* < 0.0001			
PCT < 0.1		10 (12.8)	1	1
0.1 ≤ PCT < 0.25		13 (16.7)	1.27 (0.55–2.96), *p*=0.5788	1.12 (0.47–2.68), *p*=0.7952
0.25 ≤ PCT < 0.5		8 (10.3)	1.64 (0.63–4.27), *p*=0.3131	1.60 (0.60–4.26), *p*=0.3505
PCT ≥ 0.5		47 (60.3)	4.45 (2.20–9.03), *p* < 0.0001	4.37 (2.08–9.16), *p* < 0.0001

*Bacteremia*		74 (8.7)		
PCT cut-offs	*p* < 0.0001			
PCT < 0.1		7 (9.5)	1	1
0.1 ≤ PCT < 0.25		11 (14.9)	1.54 (0.59–4.05), *p*=0.3785	1.75 (0.65–4.74), *p*=0.2711
0.25 ≤ PCT < 0.5		9 (12.2)	2.69 (0.98–7.43), *p*=0.0556	2.64 (0.92–7.59), *p*=0.0707
PCT ≥ 0.5		47 (63.5)	6.45 (2.85–14.58), *p* < 0.0001	6.38 (2.67–15.24), *p* < 0.0001
*Septic shock*		56 (6.6)		
PCT cut-offs	*p* < 0.0001			
PCT < 0.1		5 (8.9)	1	1
0.1 ≤ PCT < 0.25		3 (5.4)	0.57 (0.14–2.43), *p*=0.4514	0.50 (0.12–2.16), *p*=0.3544
0.25 ≤ PCT < 0.5		6 (10.7)	2.47 (0.74–8.26), *p*=0.1432	2.04 (0.60–6.99), *p*=0.2560
PCT ≥ 0.5		42 (75.0)	7.97 (3.10–20.51), *p* < 0.0001	6.38 (2.41–16.86), *p*=0.0002

CI, confidence interval; ICU, intensive care unit; OR, odds ratios; PCT, procalcitonin. ‡ Adjustment for gender, age, comorbidities, and infection site.

## Data Availability

Data are available upon request to the corresponding author.
